# Compressive behaviour of soft contact lenses and its effect on refractive power on the eye and handling off the eye

**DOI:** 10.1371/journal.pone.0247194

**Published:** 2021-02-19

**Authors:** Ahmad H. Shihab, Ashkan Eliasy, Bernardo T. Lopes, Richard Wu, Lynn White, Steve Jones, Brendan Geraghty, Akram Joda, Ahmed Elsheikh, Ahmed Abass

**Affiliations:** 1 Department of Mechanical, Materials and Aerospace Engineering, School of Engineering, University of Liverpool, Liverpool, United Kingdom; 2 Department of Engineering and Technology, School of Physics, Engineering & Computer Science, University of Hertfordshire, Hatfield, United Kingdom; 3 Department of Civil Engineering and Industrial Design, School of Engineering, University of Liverpool, Liverpool, United Kingdom; 4 Department of Ophthalmology, Federal University of Sao Paulo, Sao Paulo, Brazil; 5 Department of Optometry, Central Taiwan University of Science and Technology, Taichung, Taiwan; 6 R&D Department, UltraVision CLPL, Leighton Buzzard, United Kingdom; 7 Institute of Life Course and Medical Sciences, University of Liverpool, Liverpool, United Kingdom; 8 Faculty of Engineering, Higher Colleges of Technology, Dubai, UAE; 9 School of Biological Science and Biomedical Engineering, Beihang University, Beijing, China; 10 National Institute for Health Research (NIHR) Biomedical Research Centre at Moorfields Eye Hospital NHS Foundation Trust and UCL Institute of Ophthalmology, London, United Kingdom; 11 Department of Production Engineering and Mechanical Design, Faculty of Engineering, Port Said University, Port Said, Egypt; University of Michigan, UNITED STATES

## Abstract

**Purpose:**

To investigate the stress-strain behaviour of 9 soft contact lens materials, that are commonly used in the market, under uniaxial compression loading.

**Methods:**

Seven types of hydrogel and two types of silicone-hydrogel soft contact lens materials were hydrated in phosphate-buffered saline (PBS) solution then subjected to uniaxial compression loads. The load rate was set to 16.0 N/min starting with two consecutive initial 5.0 N loading cycles followed by three relaxation periods of 4.0 min within which there were two more 5.0 N loading cycles and eventually, a full loading cycle that stopped at a load of 49.0 N. The load and contraction data obtained experimentally were analysed to derive the stress-strain behaviour. Finite Element (FE) analysis was then utilised to evaluate the performance of soft contact lenses on the human eye and handling lenses off the eye.

**Results:**

Unlike tensile tests, all tested materials showed nonlinear behaviour when tested under compression. When fitted to first-order Ogden hyperelastic model, parameter μ was found to be varying in the range 0.12 to 0.74 MPa and material parameter α was found to be varying in the range 8.2 to 20.326 among the nine tested materials. Compression modulus of elasticity was 2.2 times higher than the tensile modulus of elasticity on average. FE simulation with nonlinear Ogden constitutive model showed a limited change (8%~12%) in the optical performance when compared to other material models, however, it predicted higher stress when the lens was simulated under bending during off-eye handling.

**Conclusions:**

Compression tests revealed slightly nonlinear behaviour when materials were strained under compression stress down to 15% ~ 30% of their nominal heights. Considering the physiological compression loading range of 8 mmHg, secant moduli of elasticity were 1.5% to 6.9% higher than the tension moduli of elasticity depending on the material. Tensile-based moduli of elasticity could be used in FE analysis as a step towards simulating the optical performance of soft contact lenses on-eye. However, nonlinear compression-based material models are recommended for FE analysis of soft contact lenses when lens-handling is investigated off-eye.

## Introduction

Material stiffness was not of particular interest when contact lenses first became widely commercially available [1, 2]. This is because lenses were manufactured from polymethyl methacrylate (PMMA) and various gas permeable materials that were, to all intents and purposes, rigid. Once soft contact lenses were developed by Otto Wichterle [2] in the 1960s, the physical properties and characteristics of hydrogel materials were of more interest, as they draped and moulded to the corneal shape. As a result, lenses manufactured in materials with different properties would, in theory, perform differently on the eye [1].

However, as soft contact lenses became more commercially successful, the main driver in terms of physical characteristics was oxygen permeability (DK) [3, 4] in a bid to maintain a healthy corneal metabolism. It was to this end that silicone hydrogel materials were developed, which provided higher DK values [5]. The downside of this development was that silicone hydrogel materials had much higher moduli of elasticity than hydrogels and this then brought up issues of comfort and decreased wettability, as silicon is hydrophobic [6, 7].

Since then, contact lens designers and material manufacturers have been balancing the demand from practitioners for excellent oxygen transmission with material properties that allow excellent comfort and fitting characteristics [1]. For daily disposable contact lenses, now the world’s largest selling modality, there is little the practitioner can do in terms of fit, as lenses are generally supplied with one base curve and diameter and the manufacturers have optimised the trade-off between comfort, fit and oxygen transmission. However, in the specialist contact lens market, where practitioners are fitting eyes that are outside the disposable range in terms of corneal shape and power, material stiffness is of more importance.

Such contact lenses are available in a wide variety of designs and materials, spanning hydrogels and silicone hydrogels and it is in this area where modulus becomes of interest [8]. If a practitioner changes material in order to reduce lipid deposit rates, it is useful to know how this would affect the fit of the lens and whether any adjustment is necessary. It is at this juncture that moving to a material with a similar modulus becomes important. However, in practice, soft materials with similar linear elastic moduli may not behave in the same way [1]. Thus, investigating the full range of physical characteristics and the relationship between them would be of use to specialist practitioners [9, 10].

In linear elastic materials, the modulus of elasticity (E), which is also called Young’s modulus, can be defined as the ratio of the applied stress (σ) to resulting strain (ε), [1] Eq 1.

$$E=\frac{\sigma }{\varepsilon }$$ (1)

A low modulus material is more deformable on the eye and offers less resistance against the eyelid [11]. On the other hand, a material with high modulus is comparatively stiffer and is easier to handle by wearers [12].

During normal use, a soft contact lens is subjected to bending during handling off the eye and a combination of compression due to eyelid load and shear due to tear surface tension while on the eye. Previous studies described the stress-strain behaviour of both hydrogel and silicone-hydrogel materials as linear elastic materials [13–17] based on tensile testing. However, the eyelid pressure on contact lenses cannot be described as tensile stress. Instead, it could be described as compression stress as the eyelid pushes the contact lens down towards the anterior surface of the cornea, which offers resistance due to the intraocular pressure and corneal stiffness.

Compression modulus of elasticity can be determined using the spherical indentation technique [18, 19], however, the understanding of the numerical values of the modulus of elasticity determined by indentation is underpinned by a complex combination of theoretical and experimental work [20]. Furthermore, indentation measurements consistently result in lower moduli values compared with uniaxial measurements [21], which makes indentation measurements incomparable with the moduli values that material manufacturers use to characterise their hydrogels for the contact lens industry.

Consequently, this study aimed to investigate the compressive stress-strain behaviour of 9 materials, that are commonly used in the specialist contact lens market, under uniaxial loading. The study estimates various linear and nonlinear material models for these hydrogel and silicone-hydrogel materials. In addition to the lab-based experiments, the study utilises Finite Element (FE) analysis to predict the geometry of the soft lens on the eye. Then further analysis of the resulting geometry was performed to evaluate the optical performance of these soft contact lenses. Finally, an off-eye simulation was carried out to investigate the effect of handling contact lenses made of the investigated nine materials on the maximum stress generated as a result of wearers’ Taco test that is usually carried out before each wear to check that the soft lens is not inside out.

## Materials and methods

### Uniaxial unconfined compression testing

Uniaxial compression tests were performed on nine different materials, Table 1. The material samples used in this study were provided by UltraVision CLPL (Leighton Buzzard, UK), part of the SEED group, (Tokyo, Japan).

**Table 1:** Raw data of tested materials.

Lab code	Commercial name and ISO* Classification	Base material	Manufacturer	Colour	Water Content at 20°C (%)	Oxygen permeability DK (mL O2 cm^-2^ s^-1^ mmHg^-1^)	Wet refractive index
B# 09	CONTAFLEX 77 Clear	Hydrogel	Contamac Ltd	Clear	77	45	1.3739
	filcon 2 (45) [77%]						
B# 02	DEFINITIVE (V3) 74 Blue UV	Silicone-Hydrogel	Contamac Ltd	Blue	74	60	1.3753
	efrofilcon A 5B (60) [74%]						
B# 05	DEFINITIVE (V3) 74 Clear	Silicone-Hydrogel	Contamac Ltd	Clear	74	60	1.3749
	efrofilcon A 5B (60) [74%]						
B# 03	CONTAFLEX 67 Clear	Hydrogel	Contamac Ltd	Clear	67	30	1.392
	filcon 2 (30) [67%]						
B# 06	CONTAFLEX 58 Clear	Hydrogel	Contamac Ltd	Clear	58	21	1.406
	filcon 2 (21) [58%]						
B# 08	CONTAFLEX GM3 58 Clear	Hydrogel	Contamac Ltd	Clear	58	26	1.416
	acofilcon A2 (26) [58%]						
B# 04	CONTAFLEX 55 Blue	Hydrogel	Contamac Ltd	Blue	55	19	1.4086
	methafilcon A 4 (19) [55%]						
B# 07	BENZ-G3X 49 Blue	Hydrogel	Benz Research & Development	Clear	49	15	1.425
	hioxifilcon B 1 (15) [49%]						
B# 01	CONTAFLEX 38 Clear UV	Hydrogel	Contamac Ltd	Clear	38	8	1.4381
	filcon 1 (8) [38%]						

*ISO stands for the International Organization for Standardisation.

Contact lens materials are supplied in a dry form to allow lathing, therefore, the testing procedure involved taking 6 cylindrical blanks of each material, measuring their dry dimensions before hydrating them for 8 hours in 0.9% phosphate-buffered saline (PBS) solution (Sigma Aldrich, UK). Initial hydrated dimensions were measured just before the test using a digital Vernier calliper (D00352, Duratool, Taiwan). These measurements for both length and diameter were taken at three different locations each along the sample axial and radial directions respectively and then averaged.

**Fig 1:**
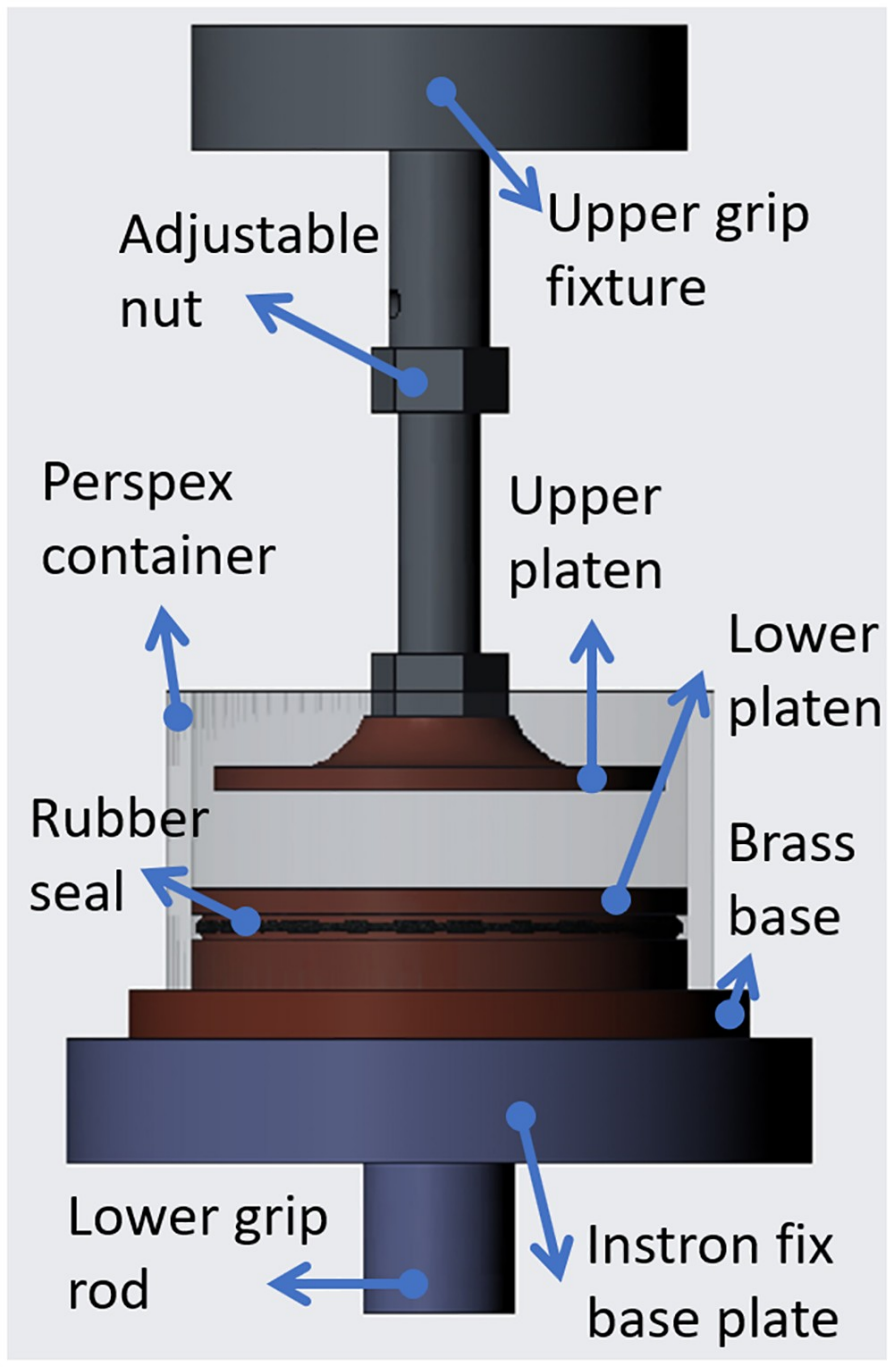
The custom-made compression test rig that was designed and manufactured at the School of Engineering, University of Liverpool, UK.

A special custom-made test rig was designed by Biomechanical Engineering Group (BioEG) using R7.0 PTC Creo software (Parametric Technology Corporation, Boston, Massachusetts, US) and manufactured at the School of Engineering, University of Liverpool, Fig 1. The rig consists of two flat platens to allow the tested samples to sit between them and a relatively heavy base to add more rigidity to the rig structure. The rig was enveloped in a perspex container to allow a hydration fluid to be used during the test. A black O-ring rubber seal was used to prevent the fluid from leaking to the base. Most of the main rig components were cut from golden coloured brass round bars which came as a 60 mm diameter raw material.

**Fig 2:**
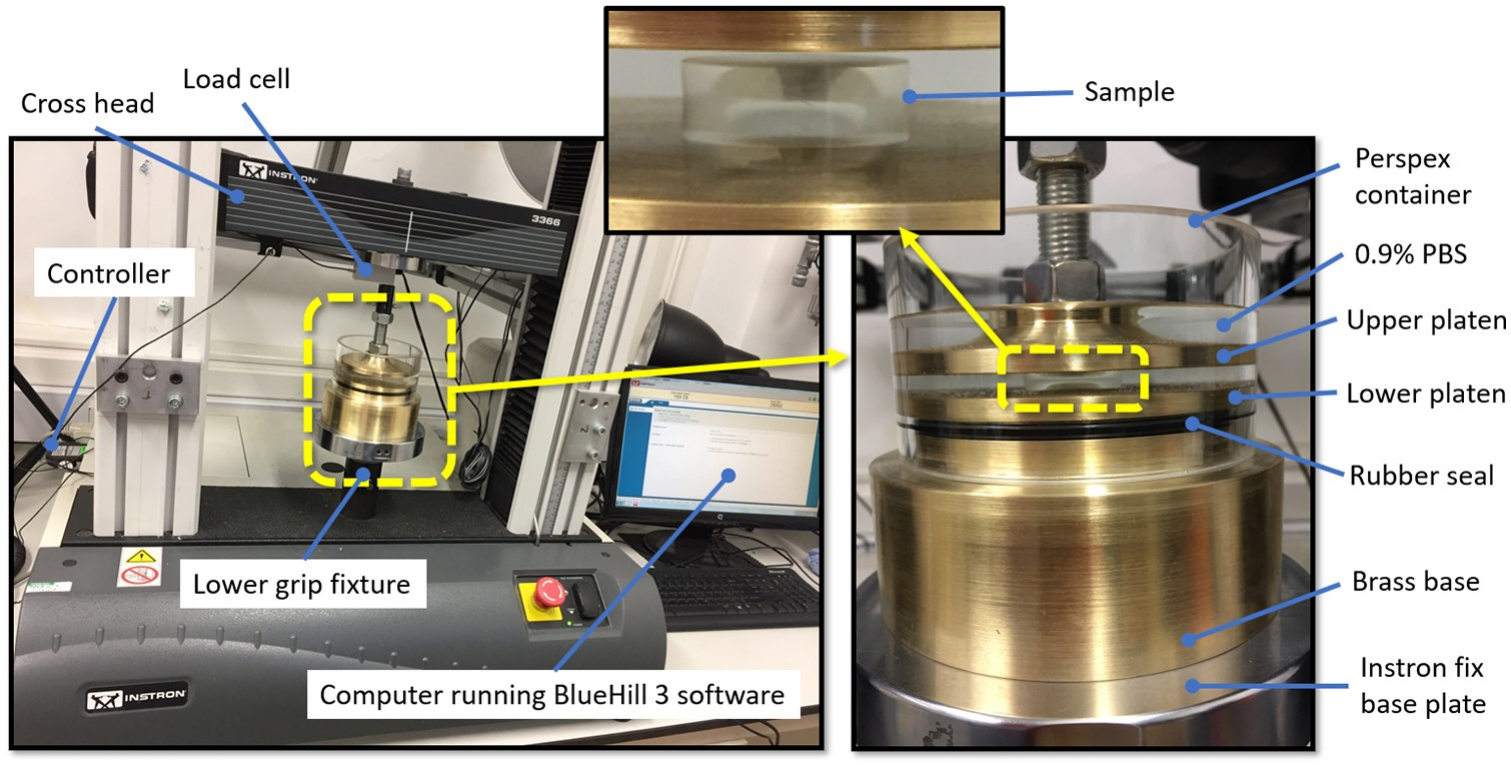
Compression test experimental setup showing the main components and the Instron machine.

The compression tests were conducted at room temperature (approx. 20°C) in the Biomechanics Laboratory at the School of Engineering, University of Liverpool (Liverpool, UK) using an Instron 3366 dual-column, table-top testing machine. This was equipped with a calibrated 50 N load cell and BlueHill 3 control software (Instron, MA, UK), Fig 2. The software allowed the design of specific test profiles and to pre-set the exact test sequence with every specimen through its automated controlled TestProfiler module. The test protocol was defined to ensure the maximum load of the loadcell is not reached.

**Fig 3:**
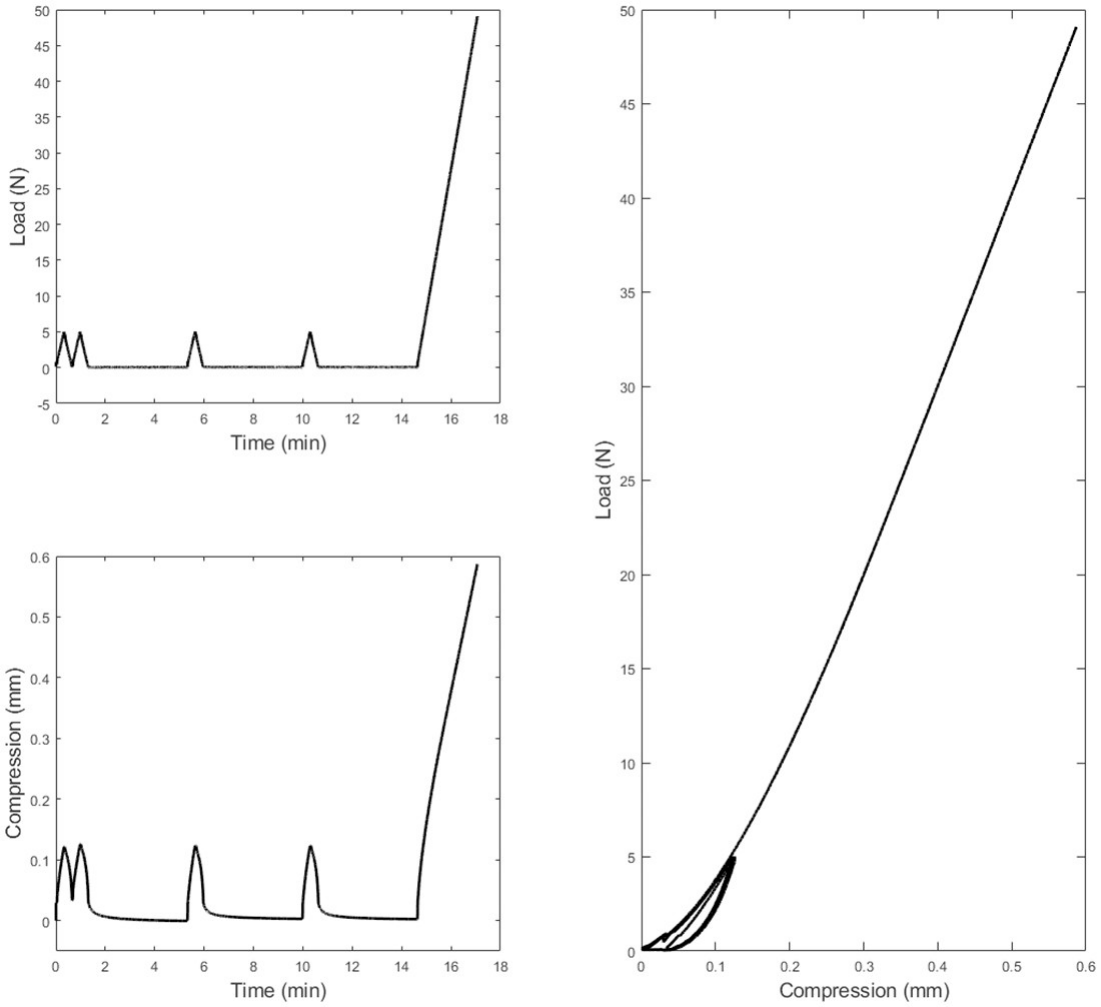
Raw compression test data as collected from the Instron BlueHill 3 control software for a hydrated Contaflex 38 Clear UV sample with 5.91 mm initial length.

Load rate was set to 16.0 N/min starting with two consecutive initial 5.0 N loading cycles followed by three relaxation periods of 4.0 min within which there were two more 5.0 N loading cycles and eventually a full loading cycle that stopped at a load 49.0 N just before the maximum loading range of the load cell, Fig 3. These values were found experimentally to be optimum for hydrogel testing under compression, enabling sufficient recovery and repeatable measurements. The samples were secured between a set of mechanical platens made of brass and specially designed for use with the Instron testing machine. A thin layer of oil-based lubricant was applied on the surfaces of the platens to reduce any restraint to the lateral sample expansion during the compression test. With the aim of maintaining hydration throughout the testing procedure, samples were submerged in a perspex chamber filled with PBS solution. The compression forces *F* at specified time increments were recorded and converted into compression stress *σ*_*E*_ values through dividing them by the hydrated samples’ initial cross-section area *A*_0_ (Eq 2) [22, 23].

$$\sigma =\frac{F}{A_{0}}$$ (2)

At the same time increments, the change in sample length Δ*L* = *L*_0_ − *L*_1_ was recorded by measuring the instantaneous length *L*_1_ and dividing Δ*L* by the initial length of the strip *L*_0_ to calculate the strain *ε* (Eq 3).

$$\varepsilon =\frac{\Delta L}{L_{0}}$$ (3)

Secant modulus of elasticity at any point (*σ*,*ε*) on the stress-strain curve can then be determined as $\frac{\sigma }{\varepsilon }$ at this point.

For nonlinear material modelling, a first-order (N = 1) Ogden hyperelastic material model [24] was used to fit and simulate the nonlinear stress-strain behaviour of the tested materials. The Ogden constitutive strain energy equation can be expressed in terms of the principal stretches as

$$U=\sum_{i=1}^{N}\frac{2\mu_{i}}{\alpha_{i}^{2}}\left({\overset{-}{\lambda }}_{1}^{\alpha_{i}}+{\overset{-}{\lambda }}_{2}^{\alpha_{i}}+{\overset{-}{\lambda }}_{3}^{\alpha_{i}}-3\right)$$ (4)

[24]

Where *U* is the strain energy; *μ*_*i*_ and *α*_*i*_, are material parameters; ${\overset{-}{\lambda }}_{i}$ are the deviatoric principal stretches (ratio between the deformed length *L*_1_ and the initial length *L*_0_) in principal directions. Since no lateral forces were applied during the compression tests conducted in the current study, principal stretches can be simplified to ${\bar{\lambda }}_{2}={\bar{\lambda }}_{3}={{\bar{\lambda }}_{1}}^{-\frac{1}{2}}$, hence

$$U=\sum_{i=1}^{N}\frac{2\mu_{i}}{\alpha_{i}^{2}}\left({\overset{-}{\lambda }}_{1}^{\alpha_{i}}+{\overset{-}{\lambda }}_{1}^{-\frac{\alpha_{i}}{2}}+{\overset{-}{\lambda }}_{1}^{-\frac{\alpha_{i}}{2}}-3\right)$$ (5)

$$U=\sum_{i=1}^{N}\frac{2\mu_{i}}{\alpha_{i}^{2}}\left({\overset{-}{\lambda }}_{1}^{\alpha_{i}}+{2\overset{-}{\lambda }}_{1}^{-\frac{\alpha_{i}}{2}}-3\right)$$ (6)

$$\sigma =\frac{\partial U}{\partial {\bar{\lambda }}_{1}}=\sum_{i=1}^{N}\frac{2\mu_{i}}{\alpha_{i}^{2}}\left({\alpha_{i}\overset{-}{\lambda }}_{1}^{\alpha_{i}}-\alpha_{i}{\overset{-}{\lambda }}_{1}^{-\frac{\alpha_{i}}{2}-1}\right)$$ (7)

where

$${\bar{\lambda }}_{1}=1+\varepsilon$$ (8)

Therefore, the stress-strain relationship can be described in uniaxial mode as:

$$\sigma =\sum_{i=1}^{N}\frac{2\mu_{i}}{\alpha_{i}}\left[{\left(1+\varepsilon \right)}^{\alpha_{i}-1}-{\left(1+\varepsilon \right)}^{-\left(\frac{\alpha_{i}}{2}+1\right)}\right]$$ (9)

[24, 25]

The parameters of the Ogden material model (Eq 9) were estimated using the particle swarm optimisation (PSO) algorithm available in MATLAB’s Global Optimization Toolbox. Lower boundaries of μ and α were set to 10^−6^ and -5 while upper boundaries were set to 10 and 50 respectively with a swarm size of 40, and a maximum number of iterations of 1000. The objective function (*err*) was set to:

$$min\left(err=\frac{1}{N}\sum_{i=1}^{N}{\left(\sigma_{exp}^{i}-\sigma_{pre}^{i}\right)}^{2}\right)$$ (10)

where *σ*_*exp*_ is the experimental stress, *σ*_*pre*_ is the model predicted stress and N is the number of strain data points. Limits of PSO optimisation were set based on the authors’ bast knowledge of inverse analysis of soft materials [13, 26–28].

#### Finite element modelling

In this study, eight-node first-order continuum solid hybrid brick elements “C3D8H” were used in two-layers of elements to the averaged eye model and soft contact lens models in ABAQUS (Dassault Systèmes, Vélizy-Villacoublay, France) FE software package licenced to the University of Liverpool, UK.

The FE mesh convergence study of the eye’s model was carried out through applying internal pressure of 15 mmHg on the internal surface of 14 eye models with node numbers varying between 804 and 750,006 nodes, half of them are in two layers, then monitoring the relevant anterior eye’s apex displacement. The outcomes showed that the number of the elements equal to 28,800 arranged in rings of 43,206 nodes in two layers has converged to the displacement of 421.14 μm at the apex node and selected as an optimal number of elements for this simulation. Likewise, the contact lenses mesh was tested by 10 Plano lenses models, five of them were in two layers with a number of nodes varying between 20166 and 53529 nodes. All contact lenses models were tested when being fitted to the selected 43206-node eye model while lenses apex displacement was recorded. The outcomes demonstrated that the model with the number of the elements equal to 30480 arranged in rings of 45969 nodes in double layers has converged to the displacement of 205.21 μm at the apex node and selected as an optimal number of elements for this simulation. During the mesh conversion study, the maximum recorded central processing unit (CPU) time for running a single model was 4.7 h in a quad-core processor 64-bit operating system.

Typically, the in-vivo human eye globe geometry is quantified whilst the eye is stressed due to the intraocular pressure (IOP) hence, geometries cannot be used directly for modelling without pre-processing. To achieve the eye’s stress-free configuration at 0 mmHg IOP, the eye globe model was initially built with the inflated dimensions, then a stress-free adjustment of the eye model was determined by following the iterative method presented in [29]. The eye’s stress-free model was computed by considering an average IOP of 15 mmHg [30] and a maximum node position error of 0.1 μm. Once the stress-free eye model was obtained, it was pressurised to 15 mmHg through a uniformly distributed static pressure on the internal surfaces of the eye globe model to mimic the aqueous and vitreous effect on the eyewall. ABAQUS nonlinear geometry option “NLGEOM” was activated during the inflation step and subsequent analysis. This option allows loads to be applied incrementally whilst updating the stiffness matrix in each increment. Hence the ABAQUS solver allows nonlinear materials to be used for certain parts without altering linear FE formulation for linear materials of other parts of the model.

The averaged eye model came as a result of secondary analysis in a collection of fully anonymised data that has been used in several previous studies [31–34] where only healthy eyes were selected to be processed. The averaged eye model’s central corneal thickness is 0.57 mm, peripheral corneal thickness is 0.63 mm, equatorial scleral thickness is 0.79 mm and posterior pole thickness 0.83 mm, S1 Data. According to the University of Liverpool’s Policy on Research Ethics, ethical approval was unnecessary for studies that solely involve the secondary analysis of fully anonymised data. Nevertheless, the study followed the tenets of the Declaration of Helsinki. Anterior eye’s topography measurements were levelled before the removal of edge artefacts following the methods detailed in our previous studies [35, 36]. Model central corneal thickness is 0.57 mm, peripheral corneal thickness is 0.63 mm, the equatorial scleral thickness is 0.79 mm and the posterior pole thickness is 0.83 mm.

**Fig 4:**
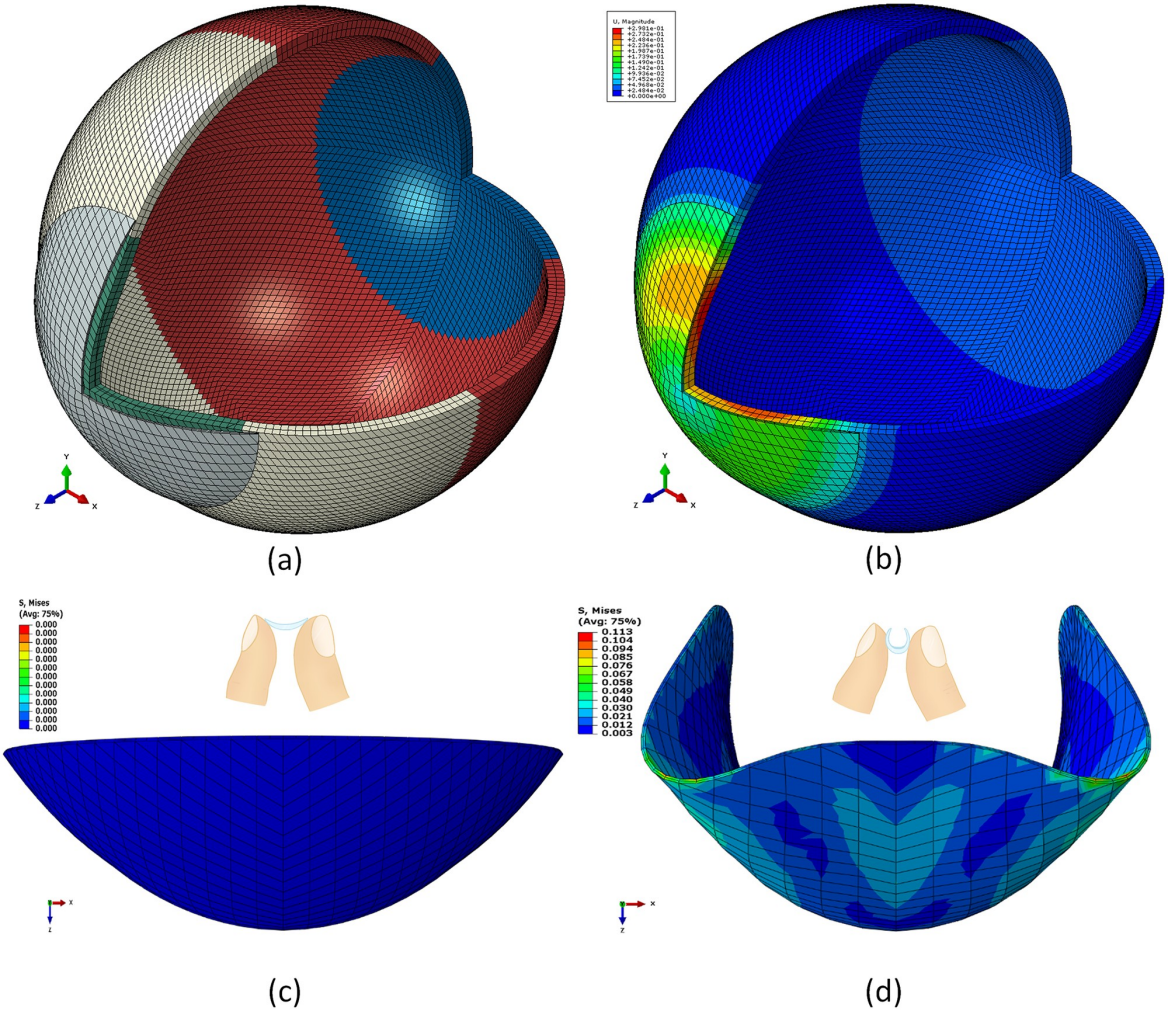
(a) Typical FE model for averaged eye and soft contact lens used in the simulation where different colours represent different material models. (b) the interaction between the soft contact lens and the eye demonstrated in displacement distribution across the anterior part of the eye. (c) typical FE model for a CONTAFLEX 77 Clear soft contact lens in no load position. (d) the stress on the soft contact lens as a result of being held between the thumb and the forefinger during handling.

In the FE simulation, Ogden material models [24] were used to represent the eye tissue’s mechanical behaviour under loading conditions following earlier experimental studies [26, 37, 38]. The eye was modelled as hyperelastic soft tissue with a water-like density of 1000 kg/m^3^ and four regions including the cornea (μ_c_ = 0.07, α_c_ = 110.8), anterior, intermediate and posterior sclera separated at elevation angles of 55°, 7.5°, -47.5° measured from the centre of the sclera [26], Fig 4a and 4b. The purpose of splitting the sclera into three regions was to consider regional mechanical properties of scleral tissue using circumferential regions of isotropic elements to replicate macroscale sclera displacements. Scleral materials were characterised as μ_s1_ = 0.441, α_s1_ = 124.5, μ_s2_ = 0.349, α_s2_ = 138.5, μ_s3_ = 0.308 and α_s3_ = 162.2.

Soft contact lens models (S2 Data) were built with four material models (based on the manufacturers’ tensile modulus of elasticity, physiological compression modulus of elasticity obtained at σ = 8 mmHg, compression moduli of elasticity obtained at ε = 0.05, and compression-based nonlinear Ogden model) for each of the nine materials included in this study. Therefore, a total of 36 soft lenses were investigated, in the simulation, on an eye model representing an averaged eye geometry.

When a soft contact lens is fitted to a cornea, it deforms [39]. Any deformation of the optic zone will affect the optical power profile. This change in lens power is termed effective power change (EPC). It is simply the refractive power of the lens post fit minus the originally designed refractive power pre-fit, which is Plano in this study. The light raytracing technique outlined in our previous study [13] was employed to measure the EPC that incurred by the fitting of each soft contact lens to the cornea. A custom-built MATLAB script performing light raytracing across the lens optic zone was written and validated using the AutoCAD software ^®^ (Autodesk, Inc., San Rafael, California, USA) [39, 40].

In the current study, a Plano (zero) powered corrective lens was used in simulating the soft contact lens’s material behaviour on the eye. By determining the lens’s power after being fitted to the eye, this power built during the fit is representing the lens’s effective power change EPC as the initial lens’s off-eye power was zero. The lens’s geometrical profile was generated via a custom-built MATLAB software before being further processed to build a FE model for the lens. The stabilisation design, commonly known as prism ballast or gravity-based stabilisation, was used in the design of the peripheral zone of soft lenses. The lens diameter was set to 14.5 mm, with base curve 8.2 mm, and central thickness 0.11 mm. Details about soft contact lens design procedures are published in our previous studies [13, 39]. Eyelid interaction was simulated by applying the eyelid pressure dynamically as a function of time. This function is based on the palpebral aperture measurement, as reported in [41]. The effect of the tear layer was simplified and simulated by applying the surface tension of the tear fluid of 43.6 mPa [42] to the back surface of the contact lens as no fluid-structure interaction analysis has been carried out in this study. Steps of the FE modelling process are listed in Table 2.

**Table 2:** Finite element simulation parameters.

Model	Step	Description	Integration scheme	Loading condition	Time
Eye	1	Stress-free iterations [29]	Implicit	Static	Normalised increments (0:1)
Eye	2	Inflation, IOP = 15 mmHg [30]	Implicit	Static	Normalised increments (0:1)
Lens on-eye	3	Eyelid pressure 8.0 mmHg [43]	Implicit	Dynamic	0.6 s, see [41]
	4	Surface tension 43.6 mPa [42]	Implicit	Static	Normalised increments (0:1)
Lens off-eye	5	Bending while held between the thumb and the forefinger’s pressure 200 Pa [44]	Implicit	Static	Normalised increments (0:1)

Soft contact lenses off-eye handling was simulated as if the lens is held between the thumb and the forefinger during a ‘Taco’ test to check if a lens is inside-out, Fig 4c and 4d. To ensure their lenses are the correct way around, wearers are usually advised to gently squeeze the lens as if they were trying to fold it in half, then look at the edge of the lens. if edges are pointing upwards and appear to meet, then the lens is in the correct way around. This test is needed before every wear as wearing a contact lens the wrong way around makes it ineffective at correcting vision (blurry vision) and may cause complications to the eye (eye pain, red eye, itchy eye) if worn inside-out for a long time. The Taco test generates bending stress on the soft lens by stretching the front surface of the lens and compresses the back surface around the bending line when the lens is being tested when it is the correct way around.

In the current study, the Taco test was simulated via a linearly increasing pressure of 200 Pa [44] applied on the external surface of the lens while the lens nodes on Y-axis were fixed. As a result, the soft lens bends, and stresses build on the lens surface. Each run ended just before any of the model iso-parametric elements got distorted by producing a negative volume. The maximum von-Mises stress was exported at this stage of simulation as the maximum possible deformation was achieved without failure.

## Results

Compression tests revealed slightly nonlinear behaviour when materials were strained under compression stress down to 15%~30% of their nominal heights. The secant modulus of elasticity E was determined for each material at the physiological loading stress 8 mmHg (10.8 kPa), which corresponds to the mean eyelid pressure as reported by Shaw et al. [43].

**Fig 5:**
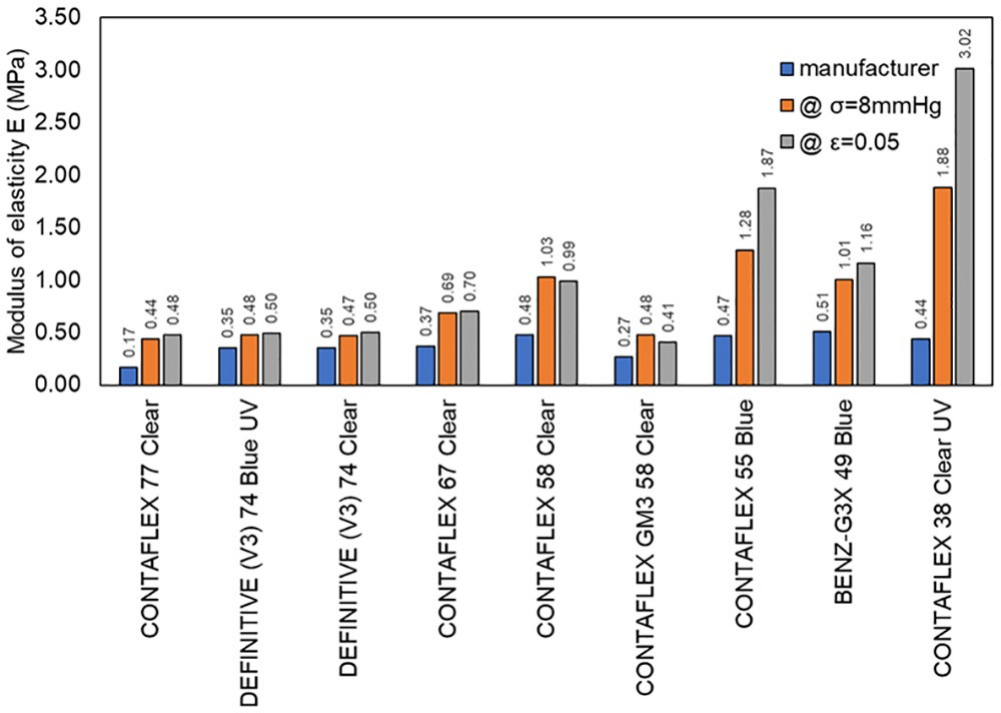
Moduli of elasticity of tested materials as reported by the manufacturer and as measured experimentally in the current study at 8 mmHg stress and 0.05 strain.

All materials tested revealed physiological compressive moduli of elasticity higher than the tensile moduli of elasticity as reported by their manufacturer with an average ratio of 2.2, Fig 5. This average ratio increased to 2.7 when moduli were calculated at ε = 0.05, Table 3.

**Table 3:** Material parameters as provided, measured, or determined.

	Load	Tension	Tension	No-load	No-load	No-load	No-load	Compression	Compression	Compression	Compression
	Data source	Manufacturer	Reported in [13]	Measured	Measured	Measured	Measured	Determined	Determined	Determined	Determined
Lab code	Commercial name \Material parameters	E (MPa)	E (MPa)	Dry length (mm)	Wet length (mm)	Dry diameter (mm)	Wet diameter (mm)	μ (MPa)	α	E (MPa) @ σ = 8mmHg	E (MPa) @ ε = 0.05
B# 09	CONTAFLEX 77 Clear	0.17	0.20	5.01±0.01	6.78±0.09	12.71±0.01	16.04±0.29	0.157	10.506	0.442	0.479
	filcon 2 (45) [77%]										
B# 02	DEFINITIVE (V3) 74 Blue UV	0.35	0.28	4.72±0.02	7.70±0.07	12.72±0.01	20.31±0.10	0.16	9.878	0.477	0.496
	efrofilcon A 5B (60) [74%]										
B# 05	DEFINITIVE (V3) 74 Clear	0.35	0.28	4.70±0.02	7.61±0.10	12.69±0.00	20.26±0.22	0.17	9.036	0.469	0.5
	efrofilcon A 5B (60) [74%]										
B# 03	CONTAFLEX 67 Clear	0.37	0.46	5.01±0.01	7.31±0.04	12.70±0.01	18.47±0.05	0.221	9.214	0.691	0.703
	filcon 2 (30) [67%]										
B# 06	CONTAFLEX 58 Clear	0.48	-	5.01±0.03	6.74±0.04	12.70±0.01	16.24±0.04	0.308	8.76	1.028	0.989
	filcon 2 (21) [58%]										
B# 08	CONTAFLEX GM3 58 Clear	0.27	-	5.01±0.01	8.05±0.06	12.71±0.01	19.18±0.06	0.122	9.93	0.481	0.406
	acofilcon A2 (26) [58%]										
B# 04	CONTAFLEX 55 Blue	0.47	-	5.03±0.00	6.02±0.03	12.77±0.00	14.07±0.10	0.718	8.185	1.284	1.872
	methafilcon A 4 (19) [55%]										
B# 07	BENZ-G3X 49 Blue	0.51	-	5.00±0.00	6.35±0.05	12.71±0.01	14.59±0.03	0.367	9.558	1.005	1.163
	hioxifilcon B 1 (15) [49%]										
B# 01	CONTAFLEX 38 Clear UV	0.44	-	5.00±0.01	5.98±0.04	12.69±0.01	13.70±0.08	0.732	20.326	1.884	3.015
	filcon 1 (8) [38%]										

**Fig 6:**
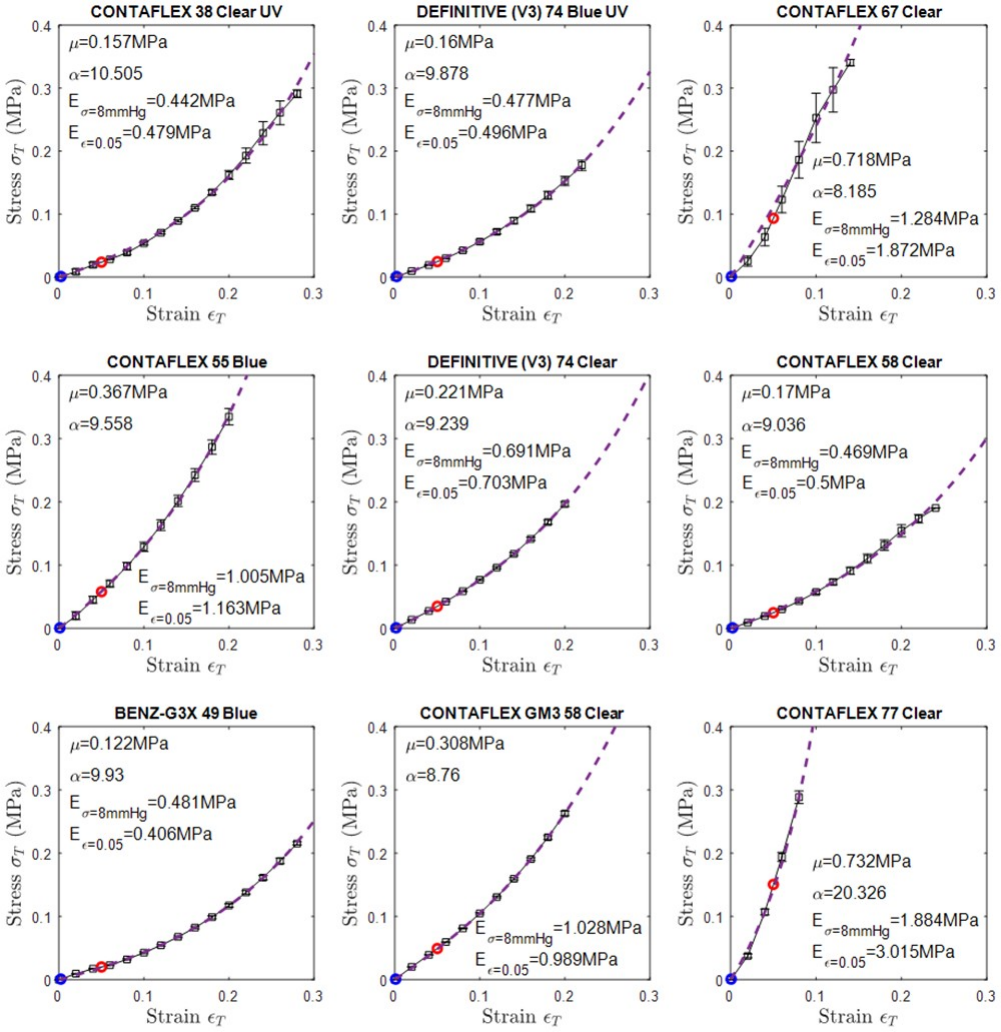
Stress *σ* verses strain *ε* for the 9 types of materials. Moduli of elasticity at 8 mmHg stress and 0.05 strain were marked in red and blue markers, respectively. The blue dashed line represents the Ogden model that fitted to the stress-strain curve.

Some of the results obtained from this study can be compared with a previous study [13] where the modulus of elasticity under tensile load was tested experimentally for four particular materials (CONTAFLEX 77 Clear, DEFINITIVE (V3) 74 Blue UV, DEFINITIVE (V3) 74 Clear, CONTAFLEX 67 Clear). The average ratio of the physiological compressive moduli of elasticity as obtained in this study to the experimentally obtained tensile moduli of elasticity reported in [13] was 1.8. Unlike the uniaxial tensile test [13], all the materials showed nonlinear behaviour when under compression, therefore when fitted to the first-order Ogden hyperelastic model [24] material parameter μ was found to be 0.33±0.22 MPa on average and material parameter α was found to be 10.6±3.5 on average, Fig 6.

**Fig 7:**
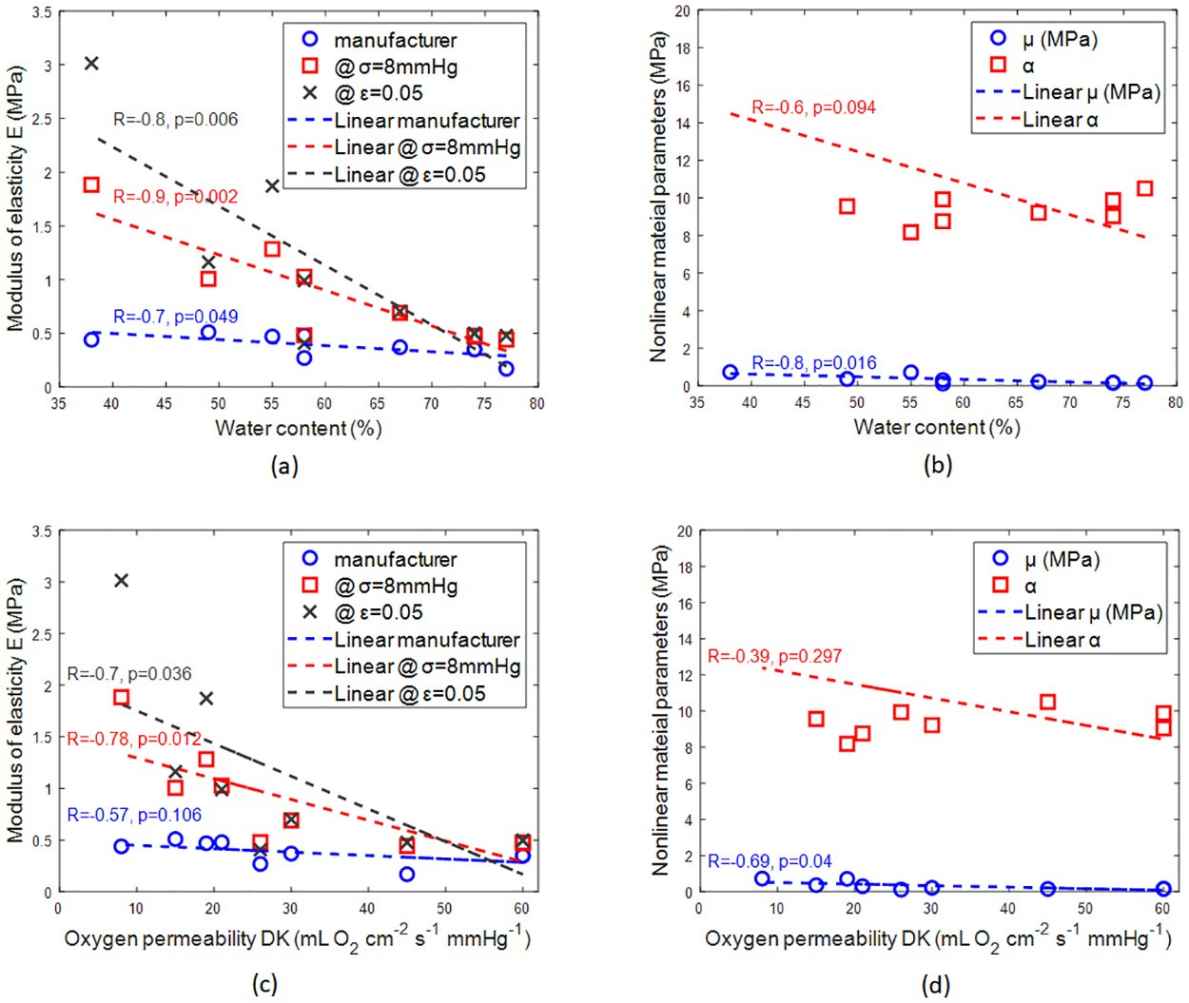
Effect of water content on the (a) moduli of elasticity, (b) nonlinear material parameters. Effect of Oxygen permeability DK on the (c) moduli of elasticity, (d) nonlinear material parameters which were used to fit the Ogden first-order model to each material.

When the stiffness of the soft material was investigated against their water contents while being hydrated, it was clear that there was a reverse linear relationship between the water content and the material stiffness. Secant moduli of elasticity in the physiological loading range (σ = 8mmHg) were strongly and inversely correlated to the water content (R = -0.88, p = 0.002), then secant moduli of elasticity at 5% strain loading range (ε = 0.05) comes second with R = -0.83, p = 0.006 and finally, the manufacturers’ moduli of elasticity with a moderate reversed linear relationship (R = -0.67, p = 0.049), Fig 7a.

The nonlinear first-order model parameters were investigated against the material water contents. The same reverse linear relationship was noted with the parameter μ (R = -0.77, p = 0.016) at a higher correlation than α (R = -0.59, p = 0.016). This indicates the overall stiffness is more significantly changing in comparison to nonlinearity behaviour of the material.

Likewise, the stiffnesses of the materials were reversely correlated with DK through moderate correlation (R = -0.57, p = 0.106) when the manufacturers’ moduli were considered, strong correlation (R = -0.78, p = 0.012) when the moduli of elasticity in the physiological loading range (σ = 8mmHg) were considered, and finally, strong correlation (R = -0.7, p = 0.036) when the moduli of elasticity 5% strain loading range (ε = 0.05) were considered Fig 7.

Nonlinear first-order model parameters were also inversely correlated with DK. Moderate reverse correlation (R = -0.69, p = 0.04) was noticed between parameter μ and DK and weak reverse correlation (R = -0.39, p = 0.297) was noticed between parameter α and DK.

In this statistical analysis, following Rumsey [45], the correlation coefficients have been interpreted as a moderate linear relationship when R = 0.5~0.69 and strong linear relationship when R = 0.7~0.99, with R = 1 indicating a perfect linear relationship. Negative R values indicate reverse linear relationships where the dependent variable on the vertical axis decreases with the increase of the independent variable on the horizontal axis.

**Fig 8:**
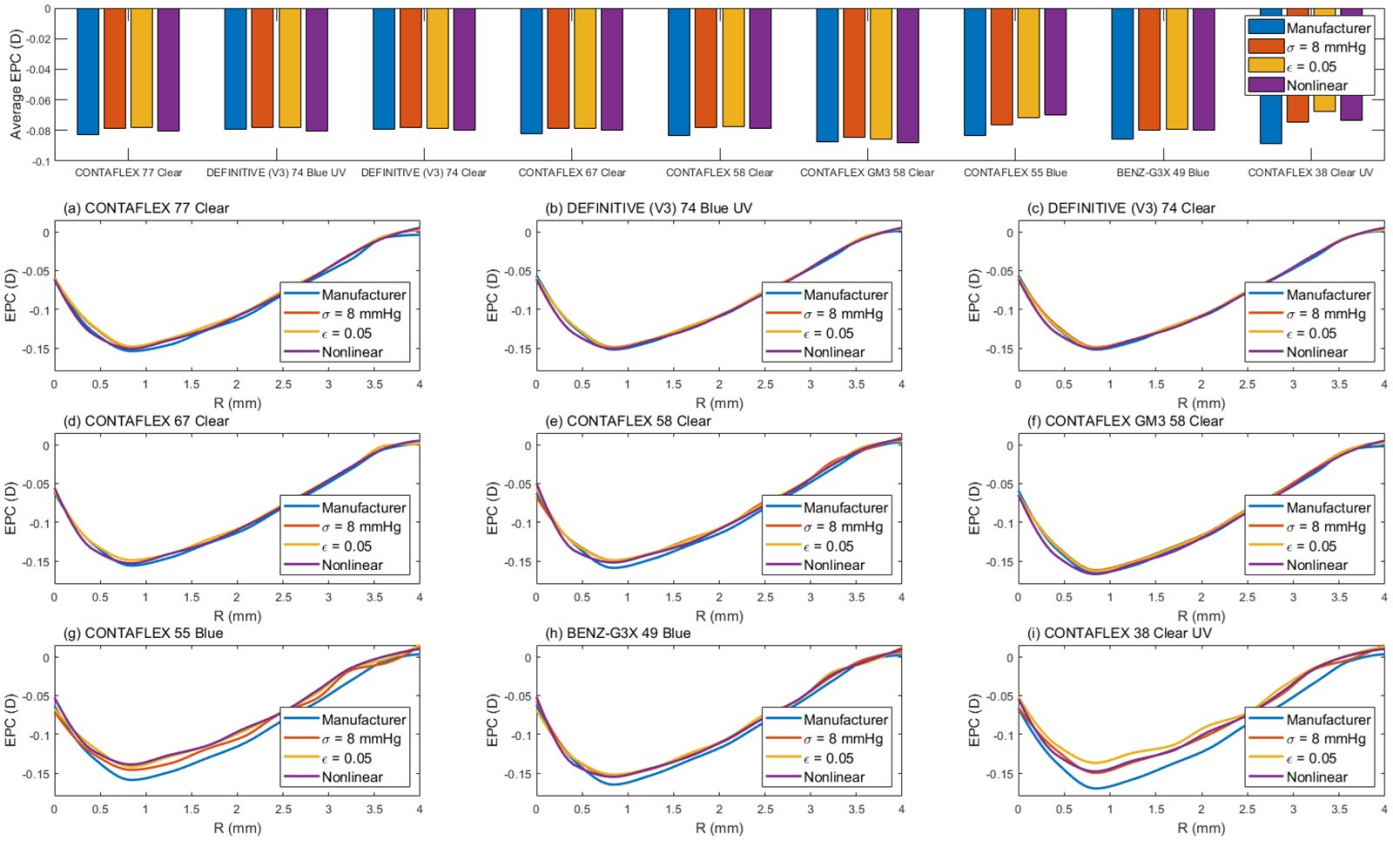
Simulated effective optical power change (EPC) of soft contact lenses models as a result of being fitted to an averaged eye model.

On one hand, in terms of the contact lenses’ performance on the eye, FE simulation showed that two of the investigated materials (CONTAFLEX 55 Blue & CONTAFLEX 38 Clear UV) are more susceptible to the type of the material model used to describe their behaviour under loading conditions than other materials, however, no significant differences were found, Fig 8. CONTAFLEX 55 Blue showed higher negative EPC at 0.8 mm radius when modelled either by the manufacturers’ modulus or the physiological modulus, while CONTAFLEX 38 Clear UV optical performance showed higher negative EPC when modelled by either the manufacturers’ modulus or the ε = 0.05 modulus. Compared to other models, the nonlinear model showed limited change in the max EPC with an average of 6% and range 8 to 12%.

**Fig 9:**
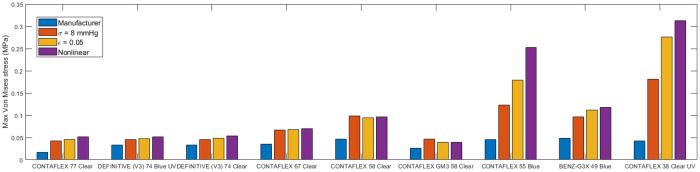
Simulated maximum von-Mises stress on the soft contact lens as a result of being held between the thumb and the forefinger during handling.

On the other hand, in terms of the contact lenses’ performance off-the-eye, Stresses generated as a result of bending during handling lenses between the thumb and the forefinger showed that stresses in the range 16.3 kPa to 312.8 kPa depending on the material and the material model used in the simulation process. Consistently, models based on the tensile moduli of elasticity were recording less von-Mises stress than the linear compression-based material models and nonlinear material models. The biggest difference in the stress caused by bending was noticed in CONTAFLEX 38 Clear UV hydrogel with von-Mises stress of 312.8 kPa when the nonlinear Ogden material model was used, compared to 42.2 kPa when the linear tensile-based material model was used. Similarly, CONTAFLEX 55 Blue recorded 252.2 kPa and 45.1 kPa with nonlinear Ogden material model compared to the linear tensile-based material model, respectively, Fig 9.

## Discussion

Currently, the stiffness of soft contact lens materials reported by their moduli of elasticity is the only way that researchers or manufacturers can evaluate material behaviours in the literature or the market. These moduli always come as a result of uniaxial tensile testing of thin strips of the material and are quite useful when evaluating the durability of the material during user handling. However, they are not accurate if they were used to predict the performance of the contact lenses on the eye. This is because, in this state, lenses are mostly subject to compressive loading.

It was clear from the results obtained from this study that the compressive stress-strain behaviour of the investigated soft contact lenses materials is nonlinear. This is evidently distinct from the relatively smooth linear stress-strain behaviour researchers reported for the same materials under tensile testing. Although it was expected that these soft materials resist compression more than tension in the physiological loading range, it was surprising that some materials like CONTAFLEX 38 Clear UV have recorded a compression modulus almost five times higher than its tensile modulus of elasticity. This indicates that such material behaviour could not be correctly predicted by the tensile modulus only. Some other investigated materials like batches of CONTAFLEX 55 Blue, CONTAFLEX 58 Clear, and BENZ-G3X 49 Blue also experienced a substantial difference (more than double) between the compressive and tensile modulus of elasticity, Table 3.

The misleading effect of using the tensile modulus of elasticity exclusively to estimate either the contact lens comfort or its optical performance on the eye can be anticipated clearly through the results of the current study. The results suggest that most contact lens materials are stiffer than it might have been estimated when they are subject to the eyelid pressure. This is a crucial finding as complications like the formation of conjunctival flaps were correlated to the stiff performance of contact lenses on the eye [46–49]. In terms of optical performance, the flexibility of soft contact lenses results in more deformation on the eye and hence possible significant alteration in dimensions and refractive powers from the desired specification [13, 39], hence poor optical performance, and failure to correct the refractive error effectively.

Water contents were always inversely correlated to the moduli of elasticity under compression regardless of the loading range, Fig 7a. The same observation is valid even when nonlinear modelling of the material behaviour was considered, Fig 7b. This indicates that the material water content could be used as an indicator to the material behaviour on the eye as it was either strongly correlated with the compressive moduli of elasticity or in the worst cases moderately correlated. On the other hand, a stiffer contact lens material may achieve excellent optical performance because of its dimensional stability on the eye but this may be achieved at the expense of user comfort as such lenses resists the eyelids’ pressure and pushes both the cornea and the eyelid with every blink. As humans blink 15 to 20 times per minute on average [50], the user’s pain accumulates during the wearing of the lens as they feel a little knock on their eye with every blink. Moreover, the fact that humans’ blink rate is several times more than required for ocular lubrication [51, 52] adds to the pain accumulation problem.

It is important here to notice the contact lenses user comfort is not exclusively dependant on the lens’s stiffness, but other factors are involved too [53]. These comfort factors include the geometric design [54] and the Oxygen permeability DK [55].

The results of DK and water content investigations showed that the nonlinear material parameter α reduce with both DK and water content, which means the material becomes more linear in behaviour while the stiffness parameter μ remained approximately the same.

On the other hand, when the soft lenses handling was investigated, the nonlinear material model was showing that the material develops maximum stress of 1.5 (DEFINITIVE (V3) 74 Blue UV) to 7.4 times (CONTAFLEX 38 Clear UV) more than the linear tensile based material model. This indicated that using compression-based material models is necessary when investigating the handling of soft contact lenses. As the nonlinear material model was giving the highest von-Mises stress among the other material models in most of the cases, considering the worst-case scenario in design nonlinear Ogden model is recommended for modelling the handling for soft contact lenses off-eye.

The study has two limitations. Since hydrogels can absorb water up to 90% of their volume or even more, their mechanical properties exhibit hyperelastic and poroelastic behaviour. The poroelasticity can be predicted by using a biphasic model [56] assuming the hydrogel is composed of a fluid phase and a solid phase. Nevertheless, in this study, the poroelasticity was not considered and the hydrogels were assumed to be composed of a single solid phase and behave as hyperelastic materials. The assumption was based on the experience that during instantaneous loading the biphasic and incompressible hyperelastic models predict equivalent stress distribution [57, 58]. Furthermore, modelling the tear film as an extra layer was not considered in this simulation, as the focus was given to the contact lens deformation in order to calculate the lens EPC.

Knowledge of commonly used contact lens materials’ behaviour under compression is essential when it comes to evaluating or optimising their performance on the ocular surface [11]. Although the contact lens market is still far from generating the ideal lens concept [59], it can benefit considerably by considering these properties in their designs. In addition, there is a need to develop comparable standardised techniques to measure soft contact lens material stiffness and other related properties as emphasised in previous studies [1, 60–62]. Unfortunately, this is currently not the case, and essential aspects of material behaviour are not considered in commercial designs [61].

## Supporting information

S1 Data Averaged eye geometry. (XLSX)

S2 Data Contact lens geometry (Plano corrective power, 14.5 mm diameter, 8.2 mm base curve, and 0.11 mm central thickness). (XLSX)
